# Photon counting detector CTA for prostate artery embolization pre-procedure planning and intra-procedural guidance

**DOI:** 10.1186/s42155-025-00567-6

**Published:** 2025-06-21

**Authors:** Paul Yousif, Forrest Linch, Prabhakar Rajiah, Jeremy D. Collins, Christopher P. Favazza, Andrea Ferrero, Michael Jundt, Scott Thompson

**Affiliations:** Department of Radiology, Mayo Clinic, Rochester, MN USA

**Keywords:** Photon Counting Detector Computed Tomography, PCD CT, Prostate artery embolization, Benign prostatic hyperplasia, Lower urinary tract symptoms, Embolization guidance

## Abstract

**Background:**

Prostate artery embolization (PAE) requires a careful understanding of pelvic arterial anatomy and identifying prostatic artery variants. Pre-procedure CTA and intra-procedural cone beam CT are traditional means of planning and performing PAE, with the latter providing guidance for embolization. Photon counting detector (PCD) CT enables ultra-high spatial resolution (UHR) whole-body imaging. For PAE, we obtain a single UHR PCD CT arterial phase acquisition, which provides both detailed pre-procedure pelvic arterial anatomic information and a dataset for 2D (angiographic) to 3D (CTA) fusion for intra-procedural guidance during PAE using embolization guidance software in the angiography suite.

**Case presentations:**

In six patients who underwent technically successful PAE via a left transradial approach, the pre-procedure diagnostic UHR pelvic PCD prostate CTA delineated bilateral prostatic artery origins and course in all cases, as confirmed with conventional angiograms. Further, registration of the UHR PCD CTA for embolization guidance was successful in all cases, augmenting vessel selection. No complication occurred.

**Conclusion:**

UHR PCD CTA is a novel imaging technology that can provide detailed prostate arterial anatomic information for pre-procedure PAE planning. Further, this same UHR PCD CTA dataset can be used for intra-procedural embolization guidance using commercially available embolization guidance software.

## Background

Prostate artery embolization (PAE) is a minimally invasive endovascular procedure included in Society of Interventional Radiology and American Urological Association guidelines for the treatment of benign prostatic hyperplasia (BPH) with moderate to severe lower urinary tract symptoms (LUTS) by international prostate symptom score (IPSS), and has a low rate of adverse effects [1–4]. The procedure consists of selective transcatheter embolization of the prostatic arterial supply with the goal of improving LUTS, as quantified with subjective (IPSS, QoL) and objective (Qmax, PVR, PSA, prostate volume) measures. PAE can be technically challenging due to variant prostatic artery origins, anastomoses with the bladder, rectum or penis, tortuosity and/or atherosclerosis of the internal iliac arteries or prostatic arteries, and acute angulation of the prostatic artery origins [5, 6].

Pre-procedure CTA or MRA can help facilitate PAE planning, including choice of arterial access site, optimal working obliquities, and choice of base catheters, microcatheters, and microwires [5, 7, 8]. Despite conventional CTAs having isotropic spatial resolution of 0.5–1 mm in each direction, small accessory or aberrant prostatic branches may not be well seen, and if unrecognized, could result in incomplete PAE [5]. MRA has similar spatial resolution limitations in delineating prostatic artery origins and distal anatomy [8]. In contrast, intra-procedural cone beam CT (CBCT) has been shown to enhance PAE efficiency and reduce overall radiation dose [9]. However, CBCT does not allow for pre-procedure planning, may be inherently limited by vessel selection (i.e. uncommon variant prostatic artery origins, such as from accessory obturator or superior rectal arteries, may not be opacified), and has inherent spatial resolution limitations. For the same reasons, small collaterals between the prostatic artery and inferior vesical, middle rectal, or internal pudendal arteries are unlikely to be demonstrated on conventional CTA or MRA, and if unrecognized at the time of PAE, non-target embolization may occur.

Ultra-high spatial resolution (UHR) photon counting detector (PCD) CT is a novel technology that directly converts X-ray energy into an electronic signal, as opposed to conventional CT detectors, which produce light in response to X-ray energy and require reflective septa. This allows UHR PCD CT to have significantly smaller detector pixel size and thus higher spatial resolution (up to 150 um in plane) than conventional CT, without loss of geometric dose efficiency or increase in radiation dose [10]. UHR PCD CT is useful in cases where higher spatial resolution adds clinical value, such as with small vessels, which has been demonstrated in coronary and periorbital vessel assessment [11].

The aim of the present study was to demonstrate the utility and feasibility of UHR PCD CTA for pre-procedural PAE planning and intra-procedural embolization guidance.

## Materials/methods

### Overview

At our institution, UHR PCD CTA (Naeotom Alpha, Siemens Healthineers) has been utilized for delineating prostate artery anatomy for PAE planning purposes. In addition, we performed 2D/3D registration with the same UHR PCD CTA for intra-procedural prostate artery embolization guidance. To do this, the pelvic PCD prostate CTA images are transferred to the control room workstation, where a subset of images spanning iliac crests through femoral heads are loaded, as further detailed below. Then, embolization guidance software (*syngo Embolization Guidance Siemens Healthineers*) [12] is used to manually trace target vessels on each side, as follows: Internal iliac artery through anterior division, parent vessel (e.g. vesicoprostatic trunk), and prostatic artery. Tracings are terminated at bifurcations of interest, such as posterior capsular supply that may also give rise to rectal arteries. Then, with the patient on the table, AP and lateral (“2D”) fluoroscopic images are registered with the UHR PCD CTA (“3D”) images. During the case, the embolization guidance software augments fluoroscopic views in real time with the traced vessel paths from the UHR PCD CTA, adjusting with obliquity and magnification [13]. Further fine tuning of the overlay may be performed in the control room using a fluoroscopic fade image from the initial steep ipsilateral oblique internal iliac angiogram. FL performed all image transfers, vessel tracings, and 2D/3D registration.

### PCD-CTA protocol

CTA examinations were performed using a photon-counting detector CT system (Naeotom Alpha, Siemens Healthineers) operating in the ultrahigh-resolution-multienergy mode (HighResUltraQuantumplus, 120 × 0.2 mm collimation) at a tube potential of 120 kV, CARE keV image quality level of 165 and pitch of 0.6 with use of CARE Dose4D. 125 mL of Iopromide 370 (Ultravist) followed by 30 mL of 0.9% saline were injected intravenously at 6 ml/second and scanning was performed from above the iliac crests through the lesser trochanter, which takes approximately 10-15 seconds. Bolus tracking starts 10 seconds after IV iodinated contrast injection with a cursor placed on the descending aorta and monitoring every 2 seconds. Once a threshold of 150 Hounsfield units is met, scanning starts after a 12 s delay. For anatomical visualization of prostate-feeding arteries and potential intra- and extra-prostatic collateral vessels, UHR PCD CTA images were reconstructed at 250 mm Field-of-view (FOV) with Bv56 kernel, quantum iterative reconstruction (QIR) strength of 2, matrix size of 1024 × 1024, slice thickness of 0.2 mm, and increment of 0.1 mm. Conversely, tracing of target arteries is performed using multienergy PCD CTA images reconstructed at 50 keV with Bv56 kernel, QIR strength of 2, matrix size of 512 × 512, slice thickness of 0.4 mm, and increment of 0.2 mm. At present, this series will not load for most patients if all images are selected, presumably due to memory constraints. We have not identified an exact upper limit based on a specific number of images, and therefore we successfully utilize the anatomic range defined above.

### PAE protocol

All PAE candidates underwent multidisciplinary evaluation with urology and vascular and interventional radiology (VIR) including consultation, IPSS/QoL assessment, uroflow ± urodynamic testing, cystoscopy, prostate volume assessment (US or MRI), prostate cancer rule-out (PSA ± prostate MRI ± biopsy), and prostate CTA using UHR PCD CTA. Prostate artery origins were categorized (Type I to V) according to the Carnevale angiographic classification [14]. A Foley catheter was placed peri-procedurally and removed at the conclusion of the case. Pre-procedure intravenous (IV) ceftriaxone was administered. All patients underwent bilateral prostatic artery embolization with 400-micron spherical embolic (HydroPearl, Terumo Corporation) via left radial artery access under moderate sedation, deep sedation, or general anesthesia as an outpatient procedure. Following radial artery sheath insertion (Glidesheath Slender, 5 French, Terumo Corporation), a 50 units per kilogram heparin bolus was administered followed by a maintenance dose of 1,000 units every 30 min thereafter. A 5 French × 135 cm base catheter (MG2, Terumo Corporation) and 1.9 French × 165 cm microcatheter (Progreat Lambda, straight tip, Terumo Corporation) were used for vessel selection along with various microwires. Arterial closure was obtained with an arterial band (TR band, Terumo Corporation). At the completion of the case, dexamethasone 4 mg IV and acetaminophen 1,000 mg IV were administered. Patients were dismissed with a 5 day course of cefdinir 300 mg by mouth twice daily, a methylprednisolone 6 day tapering dose (“Medrol dosepak,” 4 mg tablets, 6 tablets taken by mouth on day 1, decreasing by 1 tablet each day, 21 tablets total), omeprazole 20 mg by mouth once daily for 5 days, and phenazopyridine 100 mg by mouth 3 times daily as needed for painful urination up to 3 days, all starting on post-procedure day 1. For severe bladder spasms, we will provide patients trospium 20 mg by mouth twice daily, as needed. Patients were seen in the VIR outpatient clinic on post-procedure day 1 for arterial access site evaluation and a PSA check to assess biochemical response to PAE. Thereafter, patients are seen in VIR clinic at 1, 3, 6, and 12 months with IPSS/QoL evaluation and repeat PSA and uroflow at 6-months.

## Case presentations

### Case 1

Patient #1 is a 65-year-old man who presented with BPH and moderate LUTS (IPSS 16) and elected for PAE. Pre-procedure UHR PCD CTA confirmed bilateral prostatic artery origins from the obturator artery (Type III). On the right, the prostatic artery bifurcated, giving rise to an anterior central gland branch and a posterior capsular branch with candidate branches supplying the rectum. Catheter angiography with embolization guidance overlay confirmed the prostatic artery origins, as above. The left prostatic artery was selectively embolized with particles. Careful selective angiography of the right prostatic artery confirmed prostatic supply from the anterior branch, and rectal as well as posterior prostatic capsular supply from the posterior branch, as suggested by UHR PCD CTA. Each branch was selectively embolized with particles, taking special care to advance the microcatheter beyond the origin of the rectal vessels prior to injection of particles. No peri- or post-procedural complication occurred. At the 6-month follow-up visit, the patient’s IPSS had decreased to 7. 

### Case 2

Patient #2 is a 71-year-old man who presented with BPH and moderate LUTS (IPSS 14) and elected to undergo PAE. Pre-procedure UHR PCD CTA demonstrated a steep iliac artery bifurcation and tortuous external and internal iliac arteries, including an anteromedial origin of the left internal iliac artery, which then wrapped around posteriorly. Transradial access was deemed especially necessary, over transfemoral access, to minimize catheter angulation and improve microwire torque response. The bilateral prostatic arteries were shown arising from the ipsilateral pudendal arteries (Type IV). Additionally, there was candidate supply to the bladder from an accessory branch from the left prostatic artery. At catheter angiography, the left prostatic artery was selectively catheterized and supply to the bladder from the accessory branch was confirmed. This branch was coil embolized to protect the bladder during particle embolization. Subsequently, the left hemi-prostate was selectively embolized. The origin of the right vesicopudendal trunk was not clear on the initial steep ipsilateral oblique angiogram at 40 degrees. The embolization guidance overlay was used to identify a steeper right anterior obliquity of 50 degrees to better delineate the vessel origin, without performing additional digital subtraction angiography. The right prostatic artery was selectively catheterized and right hemi-prostate embolization performed. No peri- or post-procedural complication occurred. At the 3-month follow-up visit, the patient’s IPSS had decreased to 8.

### Case 3

Patient #3 is a 58-year-old man who presented with BPH and moderate LUTS (IPSS 12) and elected to undergo PAE. Pre-procedural UHR PCD CTA demonstrated the left prostatic artery arising from the anterior division of the internal iliac artery, below the vesicoprostatic trunk (Type II), and the right prostatic artery arising from the obturator artery (Type III). UHR PCD CTA demonstrated candidate rectal supply from posterior capsular branches from both the left and right prostatic arteries. At catheter angiography, the left prostatic artery was selectively catheterized. Following embolization of the anterior branch of the left prostatic artery, selective injection of the left posterior capsular branch demonstrated supply only to the rectum, therefore embolization was not performed. Subsequently, the right prostatic artery was selectively catheterized. Following embolization of the anterior branch of the right prostatic artery, selective injection of the posterior branch showed supply to the posterior prostatic capsule, but also the rectum from a proximal branch. This bifurcation was indicated by the end point of the embolization guidance overlay, and the location correlated with angiographic findings. A microcatheter was advanced distal to this rectal branch and completion embolization of the prostate was performed. No peri- or post-procedural complication occurred. At the 6-month follow-up visit, the patient’s IPSS had decreased to 4. 

### Case 4

Patient #4 is a 65-year-old man who presented with BPH and severe LUTS (IPSS 19) and elected for PAE over photo-vaporization of the prostate (PVP). Pre-procedure UHR PCD CTA demonstrated complex prostatic artery anatomy. The left and right main prostatic arteries arose from diseased trifurcations with superior and inferior vesical arteries (Type I). Accessory left prostatic branches arose from the proximal internal pudendal artery (Type IV), coursing through the seminal vesicle toward the posterior prostatic capsule, and distal obturator artery (Type V), coursing toward the anterior prostatic capsule. An accessory right prostatic artery arose from the anterior division of the right internal iliac artery (Type II), which bifurcated into separate branches with prostatic and rectal supply.

**Fig. 1:**
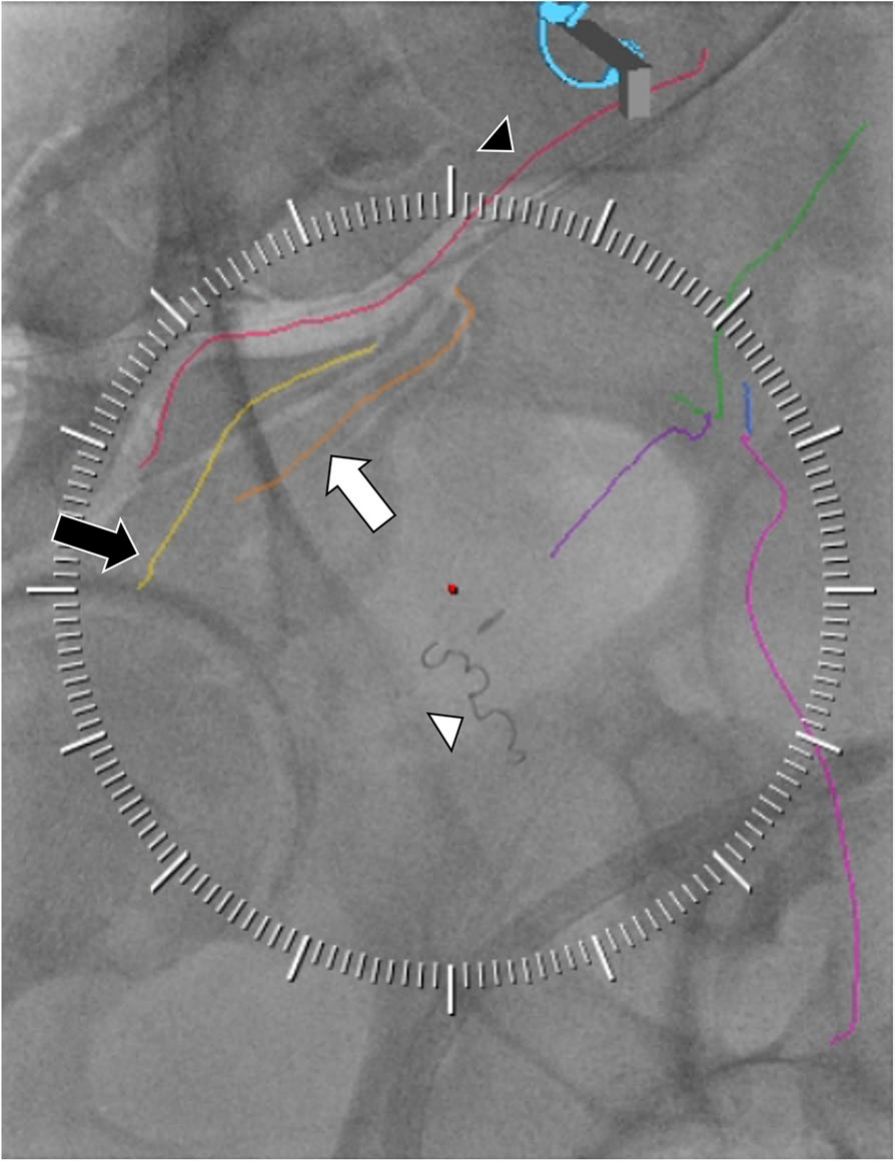
Fluoroscopic fade image of a right internal iliac artery angiogram in a steep ipsilateral oblique from patient 4 following left sided embolization, which included the use of a 2 mm x 4 cm coil (white arrowhead). Embolization guidance overlay lines traced from the PCD CTA and registered with 2D/3D fusion are shown: Red line (black arrowhead) traces the right internal iliac artery through the anterior division; Orange line (white arrow) traces the origin of the right prostatic artery from the vesicoprostatic trunk; Yellow line (black arrow) traces a right accessory prostatic vessel. The white lines arranged in a circle allow for adjustment of the overlays with the DSA image. An incidental dropped clip from prior cholecystectomy projects near the embolization coil

Catheter angiography confirmed left prostatic arteries arising from the diseased vesicoprostatic trunk (Type I) and the accessory branches from the proximal internal pudendal artery and distal obturator artery. Catheterization of the diseased left vesicoprostatic trunk was unsuccessful due to a high-grade ostial stenosis. The accessory left internal pudendal branch was catheterized and demonstrated supply to both the posterior prostatic capsule and rectum, the latter of which was not demonstrated on the pre-procedure UHR PCD CTA. Without a prostatic branch to further sub-select, this recto-prostatic branch was coil embolized for flow diversion. Catheterization of the left obturator artery accessory branch confirmed dominant supply to the left hemiprostate and was successfully embolized. Catheter angiography and embolization was subsequently performed for the main right prostatic artery arising from the vesicoprostatic trunk (Fig. 1). Next, the accessory right recto-prostatic artery was catheterized and selective injection of the posterior branch confirmed recto-prostatic anastomoses, which were coil embolized for flow diversion. No peri- or post-procedural complication occurred. At the 3-month follow-up visit, the patient’s IPSS had decreased to 6.

### Case 5

Patient #5 is a 54-year-old man who presented with BPH and severe LUTS (IPSS 26) and sought PAE to allow for discontinuation of Afluzosin (10 mg by mouth daily) due to side effects. Afluzosin is an alpha-blocker that relaxes smooth muscle and improves urinary stream, although can induce multiple side effects including orthostatic hypotension [15]. Pre-procedure UHR PCD CTA demonstrated vesicoprostatic origins (Type I) of both prostatic arteries. The embolization guidance overlay allowed for rapid orientation to the relevant anatomy for selective catheterization and embolization of the prostatic arteries. No peri- or post-procedural complication occurred. At the 3-month follow-up visit, the patient’s IPSS had decreased to 9 with IPSS QoL 2.

### Case 6

Patient #6 is a 75-year-old man who presented with BPH and severe LUTS (IPSS 31) that impacted quality of life, including the need to urinate almost hourly. Pre-procedure UHR PCD CTA demonstrated vesicoprostatic origins (Type I) of both main prostatic arteries, and a left obturator artery replaced to the inferior epigastric artery giving rise to an accessory (Type V) prostatic artery with an anastomosis to the ipsilateral Type I vessel at the prostatic capsule. Additionally, intraprostatic anastomoses observed on the UHR PCD CTA coalesced into a branch anastomosing with the distal left internal pudendal artery terminating in a penile branch. Embolization guidance overlays were especially useful in orienting the operators to the more complex left prostatic arterial supply. Additionally, special attention was given to the suspected area of penile supply, which was identified at angiography. Due to the complex intraprostatic anastomoses at the prostatic apex, gentle gelfoam embolization was performed followed by particle embolization. No peri- or post-procedural complication occurred. The patient reported significant qualitative improvement in symptoms at 4 weeks follow-up by phone, although a formal IPSS 6 months post-treatment is pending.

## Discussion

In the present series of 6 patients who underwent technically successful bilateral PAE, pre-procedure high spatial resolution (0.2–0.4 mm slice thickness) diagnostic UHR PCD CTA was successfully implemented for both PAE planning and subsequent intra-procedural embolization guidance. The pre-procedure UHR (0.2 mm) PCD CTA assisted with i) identification of the number, origin, and course of candidate prostatic arterial supply, ii) potential anastomoses with bladder, rectum or penis, and iii) informed arterial access site selection, optimal working obliquities, and choice of base catheters, microcatheters, and microwires. Additionally, 2D/3D registration of the PCD CTA with fluoroscopy using commercially available embolization guidance software was technically successful in all cases, allowing the use of embolization guidance software for intra-procedural overlays, augmenting vessel selection, which would otherwise require an intra-procedural cone beam CTA (DynaCT). DynaCT was not performed during any of our cases.

The small field of view, 0.2 mm slice thickness UHR PCD CTA images delineated all candidate prostatic artery origins and courses for selection at the time of PAE, including replaced origins and uncommon variants, and enabled anticipation of potential bladder, rectal or penile anastomoses, which were confirmed during catheter angiography. Further, in all cases, UHR PCD CTA was successfully registered for embolization guidance using a 2D/3D technique. In effect, all planning, including vessel tracing, could occur before the patient was on the table. During the procedure, embolization guidance overlays were helpful to orient operators to vessels of interest, as identified on the UHR PCD CTA. Moreover, the vessel tracing overlays adjust with fluoroscopic obliquity and magnification, without the need to perform repeat angiograms. This feature allows for troubleshooting, such as in determining the ideal obliquity for cannulation of difficult vessel angles or origins, which are a potential source of technical failure in PAE.

Such challenging PAE cases, coupled with higher body mass index (BMI), result in higher radiation exposure [16]. In our cohort (Table 1), the DAP for patients 1, 3, and 5 was within the previously published 95% confidence interval for DAP in PAE [17]. The DAP for patients 2, 4, and 6 was above this published range, which is understood by higher fluoroscopy times related to increased anatomical complexity, especially for patient 4. Higher dose accumulation occurred in patients 2 and 6, who had the first and second highest BMI (>30) in our cohort, and were also the most senior.

**Table 1:** Patient demographics and radiation parameters related to PAE and pre-procedure UHR pelvic PCD prostate CTA

Patient	1	2	3	4	5	6
Age (years)	65	71	58	65	54	75
BMI (kg/m^2^)	29.3	33.8	29.7	27.6	25.8	31.6
PAE DAP (Gy-cm^2^)	217.9	729.8	117.8	620.8	208.9	818
DSA Frames	196	166	176	239	154	170
Fluoroscopy Time (min)	41.4	61.9	21.98	79.6	32.6	63.1
PCD CTA DLP (mGy-cm)	559	594	509	466	436	605
PCD CTA Timing Prior to PAE (days)	30	2	188	133	50	1

The described application of UHR PCD CTA for intra-procedural embolization guidance has limitations. Given that the embolization guidance software was designed for use with in-room CBCT, the 2D/3D registration process described herein requires manual fine-tuning steps during the initial registration process. For improved alignment, further adjustments can be performed following a steep ipsilateral oblique conventional angiogram. Alternative methods of registration, such as 3D/3D using a non-contrast CBCT, may offer more accurate registration and reduce manual adjustment, but increase radiation exposure. An additional limitation, which affects embolization guidance software in general, is misalignment of the overlay with patient motion on the table. Further, subtle changes in anterior–posterior positioning may occur as the patient “sinks” into the fluoroscopy table pad during the procedure. Regarding the embolization guidance overlay graphics, the Siemens software renders a center line over the vessel of interest, which in the case of very small vessels, like prostatic arteries, may obscure the entire vessel and therefore wire passes, requiring toggling the overlay on or off. An alternative rendering scheme or vendor software could result in better function with respect to PAE. Presently, all of the above is well compensated for with digital subtraction angiography and fluoroscopy fades, which are correlated with the embolization guidance overlays, for vessel cannulation purposes.

Finally, while UHR PCD CTA has increased spatial resolution and excellent detection of collateral or accessory prostatic arterial supply, conventional angiography, including oblique and AP projections, remains critical for detecting potential sites of non-target embolization. Specifically, the temporal resolution of conventional angiography allows for monitoring changes in flow dynamics resulting from opening potential intra- and extra-prostatic collaterals during embolization. Further research will be necessary to determine whether routine use of UHR PCD CTA in clinical practice improves PAE efficiency and clinical outcomes while reducing overall patient and operator radiation exposure as well as procedure time. 

## Conclusion

The present series show that pre-procedure UHR PCD CTA is a novel imaging technology that provides detailed prostate arterial anatomic information for pre-procedure PAE planning and intra-procedural embolization guidance using the same dataset with commercially available embolization guidance software.

## Data Availability

All data and material available upon request.

## Abbreviations

PAE: Prostate artery embolization
BPH: Benign prostatic hyperplasia
IPSS: International prostate symptom score
CBCT: Cone Beam CT
PCD CT: Photon Counting Detector Computed Tomography
UHR: Ultra-high spatial resolution
DLP: Dose length product
DAP: Dose area product
VIR: Vascular and interventional radiology
DSA: Digital subtraction angiography
BMI: Body mass index
